# Identification of Hub Genes in Different Stages of Colorectal Cancer through an Integrated Bioinformatics Approach

**DOI:** 10.3390/ijerph18115564

**Published:** 2021-05-23

**Authors:** Abhijeet R. Patil, Ming-Ying Leung, Sourav Roy

**Affiliations:** 1 Computational Science Program, The University of Texas at El Paso, El Paso, TX 79968, USA; arpatil@miners.utep.edu (A.R.P.); mleung@utep.edu (M.-Y.L.); 2 Border Biomedical Research Center, The University of Texas at El Paso, El Paso, TX 79968, USA; 3 Department of Mathematical Sciences, The University of Texas at El Paso, El Paso, TX 79968, USA; 4 Department of Biological Sciences, The University of Texas at El Paso, El Paso, TX 79968, USA

**Keywords:** colorectal cancer, adenoma, adenocarcinoma, oxidative stress, apoptosis

## Abstract

Colorectal cancer (CRC) is the third most common cancer that contributes to cancer-related morbidity. However, the differential expression of genes in different phases of CRC is largely unknown. Moreover, very little is known about the role of stress-survival pathways in CRC. We sought to discover the hub genes and identify their roles in several key pathways, including oxidative stress and apoptosis in the different stages of CRC. To identify the hub genes that may be involved in the different stages of CRC, gene expression datasets were obtained from the gene expression omnibus (GEO) database. The differentially expressed genes (DEGs) common among the different datasets for each group were obtained using the robust rank aggregation method. Then, gene enrichment analysis was carried out with Gene Ontology and Kyoto Encyclopedia of Genes and Genomes databases. Finally, the protein-protein interaction networks were constructed using the Cytoscape software. We identified 40 hub genes and performed enrichment analysis for each group. We also used the Oncomine database to identify the DEGs related to stress-survival and apoptosis pathways involved in different stages of CRC. In conclusion, the hub genes were found to be enriched in several key pathways, including the cell cycle and p53 signaling pathway. Some of the hub genes were also reported in the stress-survival and apoptosis pathways. The hub DEGs revealed from our study may be used as biomarkers and may explain CRC development and progression mechanisms.

## 1. Introduction

Colorectal cancer (CRC) is diagnosed as the third most common cancer in males and females in the United States. It was also the third leading cause of cancer-related mortality in the year 2020 [1]. CRC has become a major health issue, with expected estimates showing 147,950 individuals to be diagnosed and 53,200 deaths to be expected from the disease in the US, in 2020 alone [1]. Between 2008 and 2017, CRC-related mortality in the younger than 55 age group increased by 1.3% per year [1]. The risk of developing CRC is contingent on certain conditions that can be categorized into environmental, lifestyle, and genetic factors [2], which concur with tumor initiation, progression, and metastasis [2,3]. Most cases of CRC start as a polyp that grows on the inner lining of the colon or rectum. Depending on their type, some polyps transform into adenocarcinoma or carcinoma. Colorectal adenocarcinomas form in the glands and can be treated through surgical or therapeutic procedures such as radiation therapy or chemotherapy. Adenomas or adenomatous polyps are pre-cancerous state polyps that may turn into carcinoma over a specific time. These adenomas can be removed surgically, and the prognosis is known to be favorable in the early stages [4]. The early detection of colorectal polyps through endoscopic screening procedure and their removal before they develop into cancers are crucial [5]. However, CRC in its early stages is often misdiagnosed as the symptoms are unusual [6] and lead to cancer metastasis, which significantly reduces the survival rate of CRC patients [4,7]. Therefore, it is of great importance to explore molecular mechanisms of CRC proliferation to improve diagnosis and treatment by understanding gene expression in CRC. The identification of multiple genes and pathways that may be involved in the occurrence and progression of CRC is also important to the development of more optimal therapeutic techniques [7,8].

With the advancement of high-throughput technologies, microarray and next-generation RNA sequencing (RNA-seq) have become popular for gene expression studies [9,10]. In this study, we have screened high-throughput gene expression data through bioinformatics approaches to identify hub genes related to CRC. Hub genes are considered to be important candidates for biomarkers in the development and progression of CRC [6]. Some previous studies have analyzed microarray data to identify hub genes in CRC [11,12,13]. However, these studies had some limitations. First, most CRC studies have considered a single microarray dataset for finding hub genes, but data from a single microarray platform might not be ideal for identifying hub genes [11,12,13]. Second, some studies that used multiple microarray datasets to find hub genes in CRC [6,7,8,14] did not compare datasets with samples from different CRC types, such as adenocarcinoma and carcinoma. There is also a lack of literature on the role of oxidative stress-induced cellular survival pathways in CRC. These limitations emphasize the necessity to utilize diverse datasets generated from different high-throughput technologies containing different tissue sample types. We analyzed the datasets to identify potential biomarkers of CRC.

In this study, we used 10 microarray datasets obtained from the gene expression omnibus (GEO) and two RNA-seq datasets from the cancer genome atlas (TCGA) to identify hub genes associated with CRC. The datasets were from different platforms such as GPL570, GPL16699, GPL4133, GPL3282, GPL15207, and Illumina HiSeq. These datasets were assigned to three groups: normal tissue vs. adenomas, normal vs. adenocarcinoma, and normal vs. carcinoma. The differentially expressed genes (DEGs) in each dataset were identified, and the robust rank aggregation (RRA) algorithm was used to integrate the gene lists across different groups. A candidate gene list with the most significantly expressed genes was generated for each group. Functional enrichment of the candidate genes in each group was analyzed using the Gene Ontology (GO) and Kyoto Encyclopedia of Genes and Genomes (KEGG) pathways. Further, protein–protein interactions were identified, and the hub genes for each group were determined through a clustering approach. The hub genes found in our study were associated with several important pathways related to CRC, such as the p53 signaling pathway. Additionally, some hub genes having a link with oxidative stress and apoptosis pathways were identified. The potential biomarkers for each group identified in our study may help promote early diagnosis and treatment of CRC.

## 2. Materials and Methods

### 2.1. Acquisition of Microarray and RNA-Seq Data

The gene expression profiles in colorectal cancer (CRC) were obtained from the gene expression omnibus (GEO) database (http://www.ncbi.nlm.nih.gov, accessed on 24 August 2020) and the cancer genome atlas (TCGA) database from the genomic data commons (GDC) data portal (https://portal.gdc.cancer.gov/, accessed on 24 August 2020). The gene expression profiles of 13 CRC datasets were divided into three groups. The first group (G1) had three datasets that consisted of colorectal adenoma (CA) samples. Group two (G2) had six datasets related to colorectal adenocarcinoma (CAC) samples. Finally, four of the colorectal carcinoma (CC) datasets were assigned to group three (G3). These datasets were chosen such that there were at least 10 samples in cases or control groups. The detailed description of the datasets used in this study is shown in Table 1.

### 2.2. Identification of Differentially Expressed Genes (DEGs) in GEO Microarray Datasets

The pre-processing step included normalization and log2 conversion of the raw matrix data for each GEO microarray dataset. All the tissue samples were based on the Affymetrix Arrays in different platforms, as shown in Table 1. The Biobase and GEOquery packages in Bioconductor R were used to load and pre-process the data. The differences in the expression levels between cases and control samples for each of the groups (CA vs. normal, CAC vs. normal, CC vs. normal) were analyzed separately using the linear models for microarray data (LIMMA) R package [26] in Bioconductor to select the statistically significant DEGs. The DEGs identified in each dataset were ranked based on the adjusted *p*-value with the Benjamini–Hochberg false discovery rate method. We defined the cut-off criteria as adjusted *p*-value < 0.05 and|FC (fold change)| > 2 to filter statistically significant DEGs in each dataset.

### 2.3. Identification of Differentially Expressed Genes (DEGs) in RNA-Seq TCGA Datasets

The RNA-seq samples of data type HTSeq-Counts were used for this analysis. The CAC samples were collected from TCGA colon adenocarcinoma (TCGA-COAD) and TCGA rectal adenocarcinoma (TCGA-READ) datasets. The TCGAbiolinks package in Bioconductor was used to obtain the data. The edgeR and LIMMA packages were used for filtering and identification of the DEGs. The lowly expressed genes were filtered out using the edgeR package. Composition biases between the libraries were eliminated using the trimmed mean of m-values (TMM) normalization. The variance modeling at the observational level (VOOM) function from the Limma package transforms the read counts into logCPMs (log Counts per million). The generalized linear model using the weighted least squares method was applied to the VOOM transformed data to test for differentially expressed genes between CAC and normal samples in the RNA-seq datasets from group G2. The threshold of adjusted *p*-value < 0.05 and |FC (fold change)| > 2 was set to filter the statistically significant DEGs in each of the RNA-seq datasets.

### 2.4. Integration of Ranked Lists of Differentially Expressed Genes (DEGs) in Groups G1, G2, and G3

Each dataset had a different number of DEGs (Table 1). We obtained three ranked lists of DEGs for three datasets in G1, six ranked lists of DEGs for G2, and four ranked DEG lists for G3. Next, to integrate the different lists of DEGs into a robust individual list of DEGs for each group, we applied the robust rank aggregation (RRA) algorithm [27]. The ranked lists of DEGs in a group were considered, and for each gene, the RRA algorithm looked at how the gene was positioned in the ranked lists of DEGs and compared this to the baseline case where all the preference lists were randomly shuffled [27]. Finally, a *p*-value was assigned for all the genes. This showed how much better the genes were positioned in the ranked lists than expected by chance. The *p*-value for each gene showed its significance in the final robust ranked list.

Overall, the RRA algorithm is based on a probabilistic model for aggregating the genes from different datasets into a final robust ranked list. This method is computationally efficient and statistically robust. The “RobustRankAggreg” package in R was used to perform the gene integration in groups G1, G2, and G3.

### 2.5. Functional and Pathway Enrichment Analysis

The database for annotation, visualization, and integrated discovery (DAVID, https://david.ncifcrf.gov/home.jsp, version 6.8, accessed on 15 November 2020) is a publicly available bioinformatics database. This database helps to identify various biological pathways of DEGs through a set of functional annotation tools. Gene ontology (GO, http://geneontology.org/, accessed on 15 November 2020) classifies the description of gene function into three categories: biological process (BP), cellular component (CC), and molecular function (MF). The Kyoto Encyclopedia of Genes and Genomes (KEGG, https://www.genome.jp/kegg/, accessed on 15 November 2020) database resource provides an understanding of high-level gene functions and biological signaling pathways. The GO term and KEGG pathway enrichment analyses were performed with the help of the DAVID database. The terms with *p* < 0.05 were considered to be statistically significant.

### 2.6. Protein–Protein Interaction

STRINGdb (Search Tool for the Retrieval of Interacting proteins database, https://string-db.org/, accessed on 16 January 2021) version 11.0 was used to identify various protein–protein interaction (PPI) networks of DEGs based on a confidence score set to medium = 0.7. To analyze the PPI network from Stringdb, Cytoscape (https://cytoscape.org/, accessed on 16 January 2021), an open-source bioinformatics software for loading, visualizing, and integrating complex PPI networks, was used. The StringApp plugin in Cytoscape was used to load the PPI network from Stringdb, and the network analyzer plugin was applied to measure the degree of interaction between nodes, and the network with upregulated and downregulated genes was displayed. The MCODE plugin was also applied in Cytoscape to analyze the network further. MCODE uses a clustering algorithm to generate the network clusters to find the densely connected regions. The key clusters in the network were filtered with degree cutoff = 2, node score cut off = 0.2, k-core = 2, and max. depth = 100.

### 2.7. Hub Gene Screening and Analysis

We used the network analyzer algorithm to compute the number of connected pairs of nodes, the average number of neighbors, and node degrees. The node degree represents the interaction score assigned for each gene. The genes were ranked based on the degree of interactions among them. The DEGs with the highest degree of interaction are defined as hub genes [28,29]. We determined the hub genes (top 40 DEGs) from each group ranked based on the highest degree of interaction, using the network analyzer results. Functional enrichment of hub genes was further analyzed and noted. We also used the MCODE algorithm to verify the hub gene results further. The MCODE algorithm grouped the genes from the original network into modules and ranked them based on degrees. The top two clusters were selected from each of the groups to display the interactions.

### 2.8. Identification of Oxidative Stress-Response and Apoptosis-Associated Genes in CRC with Oncomine

The Oncomine research premium edition (https://www.oncomine.org/, accessed on 7 May 2020) was used to analyze the genes related to oxidative stress and apoptosis survival pathways. The datasets in Oncomine were grouped into three groups similar to the entire CRC dataset. As shown in Table 2, G1, G2, and G3 contain the CA, CAC, and CC samples, respectively. The concept-based filters for response to oxidative stress and apoptosis were applied separately on the datasets to find the DEGs related to each concept. The threshold of adjusted *p*-value < 0.05 and |FC (fold change)| > 2 was set to filter the statistically significant DEGs related to oxidative stress and apoptosis.

## 3. Results

### 3.1. Genes Differentially Expressed in Colorectal Adenoma (G1) Datasets

The first group (G1) consisted of three datasets that compared expression between normal and colorectal adenoma (CA) samples. These datasets contained 64 (GSE8671), 69 (GSE20916), and 80 (GSE89076) samples, respectively (Table 1). There were 50% to 65% cases and 50% to 45% control samples, respectively. The number of genes for which data was available ranged from ~27,700 to ~58,700 (Table 1). The DEGs were identified using the Limma procedure for all the datasets. There were 5498, 2761, and 7538 DEGs detected in the GSE8671, GSE20916, and GSE89076 datasets, respectively (Table 1). For each dataset, there were more genes that were significantly downregulated (DR) in the CA samples than those that were upregulated (UR) (Table 1).

The RRA method in R was used to perform gene integration across all G1 datasets. The *p*-value cut-off was set to 0.05 to filter the ranked DEGs. The number of robust DEGs detected for the G1 was 635, out of which 186 were UR, and 449 were DR (Table 3).

### 3.2. Genes Differentially Expressed in Colorectal Adenocarcinoma (G2) Datasets

In the second group (G2), there were six datasets with normal and colorectal adenocarcinoma (CAC) samples. The number of samples in these datasets ranged from ~35 to ~600 (Table 1). The number of cases was more when compared to control in all the datasets. The LIMMA procedure was used to find the DEGs in the GSE20842, GSE20916, GSE39582, and GSE110225 microarray datasets. The LIMMA-VOOM approach was used to find DEGs in RNA-seq datasets such as TCGA-COAD and TCGA-READ. There were ~1100 to ~6800 DEGs identified in the G2 datasets (Table 1), with the count of DR genes being relatively higher than UR genes. The RRA method was applied to integrate the genes from all the G2 datasets. The number of robust UR and DR genes in G2 were 499 and 494, respectively, adding up to a total of 993 DEGs, as shown in Table 3.

### 3.3. Genes Differentially Expressed in Colorectal Carcinoma (G3) Datasets

The third group (G3) consisted of four datasets that compared expression between normal and colorectal carcinoma (CC) samples. These datasets contained 30 (GSE3964), 28 (GSE113513), 44 (GSE32323), and 148 (GSE21510) samples, respectively (Table 1). GSE113513 had the same number of cases and controls (Table 1). The DEGs were identified using the Limma procedure for all the datasets. Totals of 483, 2864, 4671, and 7720 DEGs were detected in the GSE3964, GSE113513, GSE32323, and GSE21510 datasets, respectively (Table 1). More DR genes than UR genes were found in GSE3964 and GSE113513. However, in the other two datasets, GSE32323 and GSE21510, the UR genes outnumbered DR genes (Table 1). The RRA approach in R was used to perform gene integration across all G3 datasets. The number of robust DEGs detected for the G3 was 285, out of which 206 were UR, and 79 were DR (Table 3).

Finally, we generated a heatmap to illustrate the change in expression levels of overlapping robust DEGs across all three groups (Figure S1). We could detect changes in the expression patterns between adenoma, adenocarcinoma, and carcinoma groups. Some of the DEGs that had lower expression in adenocarcinoma were significantly increased in carcinoma datasets.

### 3.4. GO Term Enrichment Analysis

The ranked RRA DEGs for each group, G1, G2, and G3, including both UR and DR genes, were submitted separately to DAVID to retrieve the overrepresented GO categories and KEGG pathways.

#### 3.4.1. GO Terms and KEGG Pathway Enrichment Analysis of UR Genes

**Figure 1:**
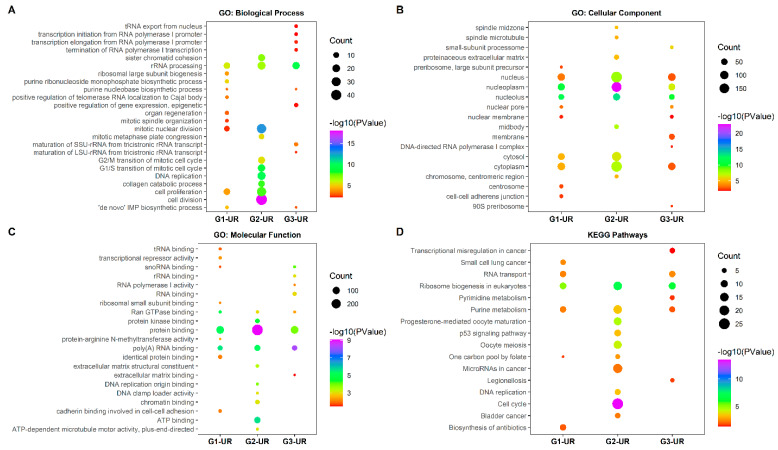
GO and KEGG pathway enrichment of RRA-UR genes. (**A**) GO enrichment of biological process. (**B**) GO enrichment of cellular components. (**C**) GO enrichment of molecular function. (**D**) KEGG pathway enrichment. Abbreviations: UR, Upregulated; GO, gene ontology; KEGG, Kyoto encyclopedia of genes and genomes.

First, we wanted to check for the enrichment of the top 10 GO classifications and KEGG pathways for the UR genes within all three groups (Figure 1). Among the top 10 subcategories shown, the top three for each group are described here. In G1-UR, the top three GO biological processes were rRNA processing, purine ribonucleoside monophosphate biosynthetic process, and "de novo" IMP biosynthetic process (Figure 1A). The GO biological processes in G2 were cell division, mitotic nuclear division, and DNA replication. Finally, for G3, the UR genes were associated with rRNA processing, maturation of SSU-rRNA from tricistronic rRNA transcript, and purine nucleobase biosynthetic process (Figure 1A). Overall, for each group the top 10 sub-categories were found to be different, with only a few overlaps. For example, the G1/S transition of the mitotic cell cycle, G1/M transition of the mitotic cell cycle, and cell division were found within the top 10 pathways of G2 and were not significantly enriched in other groups. The number of UR genes in cell proliferation increased from 20 in G1 to 30 in G2. The rRNA processing was common in all three groups, with almost the same count of UR genes. However, the *p*-value was lowest in G3 compared to G1 and G2, showing a higher level of significance.

The comparison of the 10 cellular component ontologies in G1, G2, and G3 is shown in Figure 1. The nucleolus and nucleoplasm are the common GO cellular components in all the groups, and they were also the most significant pathways in each group. The nucleus, nucleoplasm, and cytoplasm had more UR genes involved from G2 when compared to the other groups (Figure 1B). There was also an increase in the number of UR genes and the enrichment in cytosol from G1 to G2.

In the molecular function analysis, the G1-UR genes were significantly enriched in poly(A) RNA binding, protein binding, Ran GTPase binding, and protein-arginine N-methyltransferase activity. The G2-UR were found to be enriched in protein binding, ATP binding, and poly(A) RNA binding. The G3-UR were enriched in poly(A) RNA binding, snoRNA binding, and protein binding (Figure 1C). We observed that the poly(A) RNA binding and protein binding were common in all groups. The *p*-value for poly(A) RNA binding suggested an increase in the enrichment of poly(A) RNA binding from G1 to G3. More than 100 of the UR genes were involved in protein binding, with the highest number of UR genes from G2. We also observed some of the unique molecular functions within the top 10 reported in each group. The cadherin binding involved in cell-cell adhesion, tRNA binding, and transcriptional repressor activity was only found in G1 and not in G2 or G3. The ATP binding and chromatin binding were unique to G2. The unique functions in G3 were rRNA binding, RNA polymerase-I activity, and RNA binding.

The KEGG pathway analysis was performed to gain further insights into the identified UR genes. The top three KEGG pathways from G1 were significantly enriched in ribosome biogenesis in eukaryotes, small cell lung cancer, and RNA transport (Figure 1D). The UR genes in G2 were found to be enriched in the cell cycle, ribosome biogenesis, and oocyte meiosis in eukaryotes. Finally, the UR genes in G3 were found in ribosome biogenesis in eukaryotes, RNA transport, and purine metabolism. The ribosome biogenesis pathway was found in all the groups; however, it was highly enriched in G2. Several pathways were enriched only in G2 (colorectal adenocarcinoma), such as the p53 signaling pathway, cell cycle, microRNAs in cancer, and DNA replication. Transcriptional misregulation in the cancer pathway was found only in G3, the carcinoma group.

#### 3.4.2. GO Terms and KEGG Pathway Enrichment Analysis of DR Genes

**Figure 2:**
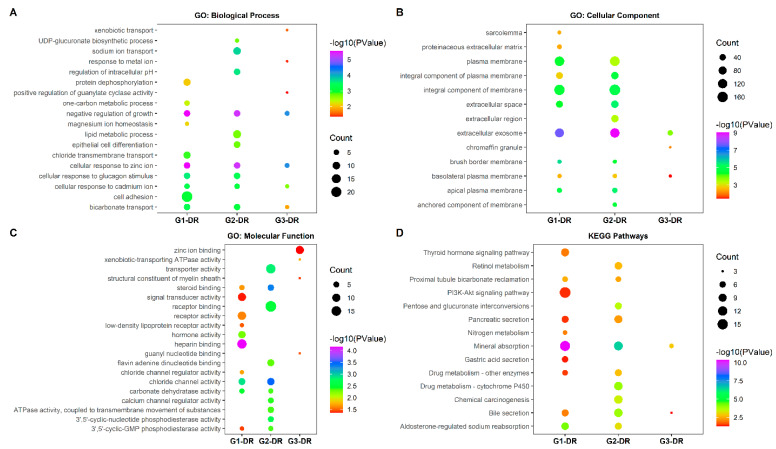
GO and KEGG pathway enrichment of RRA DR genes. (**A**) GO enrichment of biological process. (**B**) GO enrichment of cellular components. (**C**) GO enrichment of molecular function. (**D**) KEGG pathway enrichment. Abbreviations: DR, downregulated.

The summary of GO subcategories and KEGG pathways for the DR genes in the top 10 pathways based on the *p*-values in G1, G2, and G3 is shown in Figure 2. The top three GO subtypes and KEGG pathways for groups G1, G2, and G3 are discussed here. In the biological process, the DR genes in the top 10 pathways based on *p*-values in G1, G2, and G3 genes are shown in Figure 2A. The top three pathways in G1 were cellular response to zinc ion, negative regulation of growth, and cellular response to glucagon stimulus. The negative regulation of growth, response to zinc ion, and sodium ion transport were the top three processes in G2. In G3, the negative regulation of growth, cellular response to zinc ion, and cellular response to cadmium ion were highly enriched. We observed that the negative regulation of growth and response to zinc ion were shared among all three groups, and there was an increase in enrichment from G1 to G3. There were also some vital pathways, such as cell adhesion and protein dephosphorylation, that were found in G1 but not within the top 10 processes in the other two groups.

In the GO cellular component for G1, the extracellular exosome, brush border membrane, and apical plasma membrane were found to be the top significant pathways. The extracellular exosome, extracellular space, and apical plasma membrane were the top enriched pathways where the DR genes were involved in G2 (Figure 2B). The extracellular exosome, chromaffin granule, and basolateral plasma membrane were the top GO cellular signals in G3. The integral component of the membrane appeared to be highly enriched in G2-DR compared to G1-DR.

In the molecular function part (Figure 2C), the top three pathways for G1-DR were heparin-binding, chloride channel activity, and carbonate dehydratase activity. The top three for G2-DR were in chloride channel activity, steroid-binding, and transporter activity, and for the G3-DR, in xenobiotic-transporting ATPase activity, guanyl nucleotide-binding, and structural constituent of the myelin sheath. There were no overlaps within the top 10 molecular functions among all groups. However, the enrichment of several functions such as steroid binding, chloride channel activity, carbonate dehydrate activity, and 3′,5′-cyclic-GMP phosphodiesterase activity was observed to increase from G1 to G2.

The KEGG pathways for the DR genes (Figure 2D) in G1 were mainly enriched in mineral absorption, aldosterone-regulated sodium reabsorption, and proximal tubule bicarbonate reclamation. In G2, the DR genes were significantly enriched in mineral absorption, pentose and glucuronate interconversions, and drug metabolism-cytochrome P450. The DR genes in G3 were enriched in mineral absorption and bile secretion. The mineral absorption and bile secretion were commonly found in all groups. However, the number of DR genes involved and the corresponding *p*-value in these pathways decreased from G1 to G3. There were several unique KEGG pathways observed in each group. Gastric acid secretion, thyroid hormone signaling pathway, and P13K-Akt signaling pathway were enriched only in G1. Similarly, in G2, drug metabolism- cytochrome P450 and chemical carcinogenesis were enriched.

**Figure 3:**
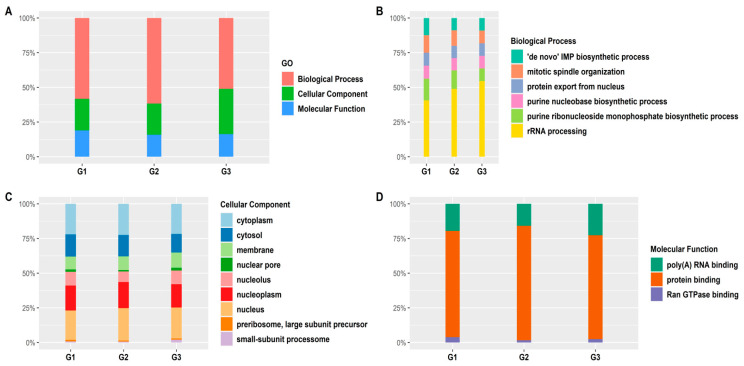
Functional group enrichment analysis of UR RRA genes in G1, G2, and G3. (**A**) Analyses of GO functional groups within G1, G2, and G3. The Y-axis of these 100% stacked columns shows the percentage of genes that fall within each GO functional group. (**B**–**D**) Common GO functionalities between all groups. (**B**) GO biological processes. (**C**) Molecular functions. (**D**) Cellular components.

After identifying the top 10 GO terms in each group, we further showed the variation of enrichment for commonly found GO terms in G1, G2, and G3. The summary of GO term enrichment analysis for the RRA UR genes is shown in Figure 3. First, we checked the percentage of genes present in different GO terms such as biological processes, cellular components, and molecular function in all the groups (Figure 3A). We observed that the percentage of genes involved in biological processes in G3 was lower than that in G1 and G2. The count of UR genes involved in molecular function decreased slightly from G1 to G3. On the other hand, the count of genes associated with cellular components increased slightly from G1 to G3. To analyze the similarities and differences in the percentage of genes enriched within each category, we looked at the overlapping sub-categories of all the groups.

The GO terms for common biological processes for G1, G2, and G3 UR genes were checked. We found that rRNA processing had the highest share in each group, increasing from G1 to G3. The percentage of genes involved in mitotic spindle organization, de novo IMP biosynthetic processes, and purine ribonucleotide monophosphate biosynthetic processes decreased from G1 to G3 (Figure 3B). The summary of common cellular component pathways is shown in Figure 3C. We did not see a significant change in the percentage of genes involved in many of the components except for cytosol, which decreased slightly from G1 to G3. No significant changes were observed in the common molecular function enrichment analysis. Similar numbers of genes from each of the three groups were enriched in three overlapping sub-categories of molecular functions (Figure 3D).

**Figure 4:**
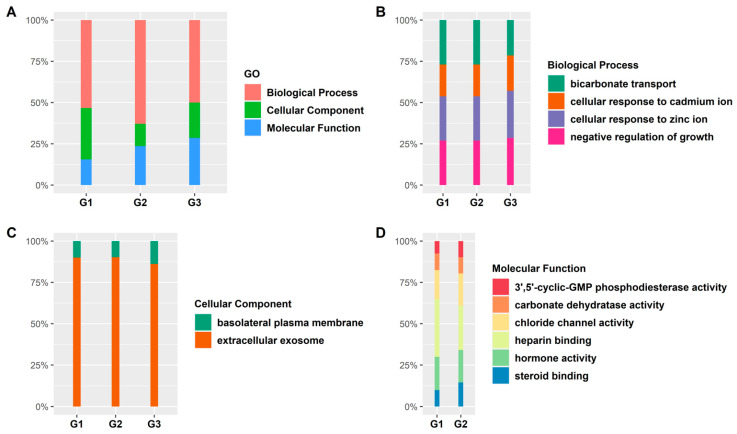
Functional group enrichment analysis of DR RRA genes in G1, G2, and G3. (**A**) Analyses of GO functional groups within G1, G2, and G3. The Y-axis of these 100% stacked columns shows the percentage of genes that fall within each GO functional group. (**B**–**D**) Common GO functionalities between all groups. (**B**) Biological processes. (**C**) Molecular functions. (**D**) Cellular components.

We developed a summary of GO term enrichment analysis for the RRA DR genes (Figure 4). The overall summary of the percentage of genes present in the GO biological processes, GO molecular functions, and GO cellular components in G1, G2, and G3 is shown in Figure 4A.

Among the common pathways found in the GO biological processes, we observed that the cellular response to cadmium ion, cellular response to zinc ion, and negative regulation of growth had an increased share of DR genes from G1 to G3. However, the quantity of DR genes in bicarbonate transport dropped from G1 to G3 (Figure 4B). Next, we looked at the common pathways present in GO cellular components (Figure 4B). Only two common cellular components were found where the percentage of DR genes in the basolateral plasma membrane displayed an increase from G1 to G3. The percentage of DR genes in the extracellular exosome decreased (Figure 4C). Finally, we examined for enrichment in sub-categories within molecular function among the three groups. However, G3 did not have any overlaps with the other two groups. Therefore, only G1 and G2 are shown in Figure 4D. The percentage of DR genes in 3′,5′-cyclic-GMP phosphodiesterase activity, steroid binding, and hormone activity increased from G1 to G2 (Figure 4D).

### 3.5. Protein-Protein Interaction (PPI) Network Construction and Module Selection for Key Genes

#### 3.5.1. PPI Network Construction and Module Selection in G1, G2, and G3

PPI networks of DEGs in G1, G2, and G3 were constructed with the string app in Cytoscape. The original PPI network developed for each group had many nodes and edges among them. The nodes represent the proteins, and the edges are the interactions. The PPI network for G1 had 615 nodes and 846 edges (Figure S2). The PPI network for G2 had 816 nodes and 3056 edges (Figure S3). Finally, for G3, the PPI network had 280 nodes and 545 edges (Figure S4). As seen in these figures, the networks are very dense, and it is very difficult to interpret important information from these interaction networks. Therefore, we applied the network analyzer algorithm to filter the DEGs with a high degree of interaction score. The top 40 DEGs with high scores were determined as hub genes in each group, similar to that in studies by Nangraj et al. (2020) and Hu et al. (2020). To further validate our results, we applied the MCODE algorithm to divide the original dense networks into modules. The top two clusters with high scores in each group were considered to be significant.

**Figure 5:**
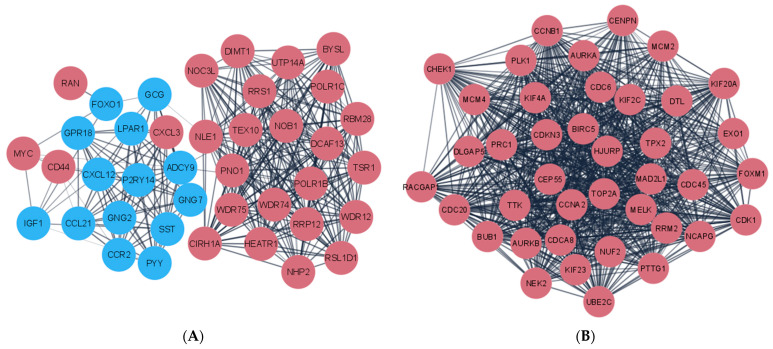

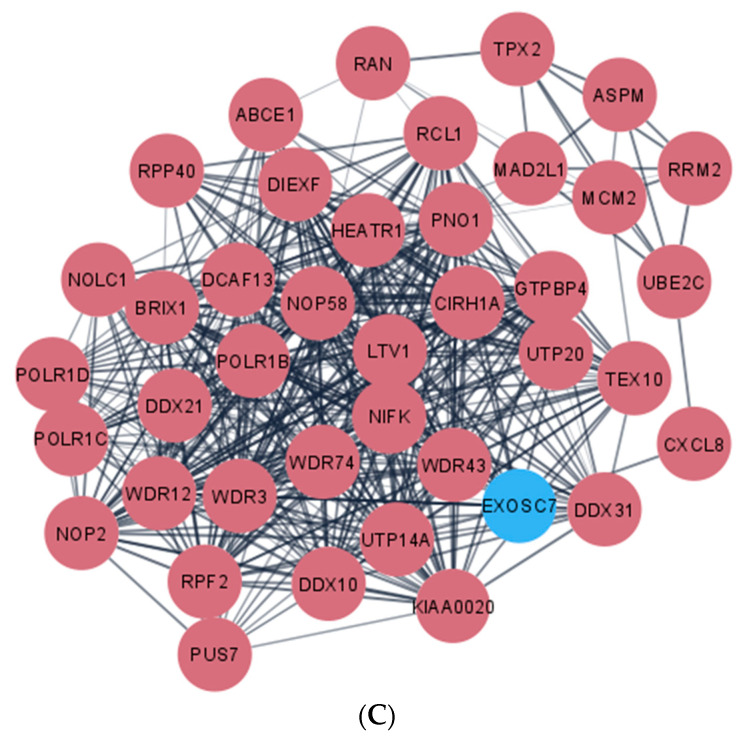
(**A**) Top 40 hub genes in G1. (**B**) Hub genes in G2. (**C**) Hub genes in G3. Dark red color—UR; Blue color—DR.

The top 40 genes with the highest degree of interaction from the original dense networks in each group were termed hub genes and are reported in Table S1; the PPI networks of hub genes in G1, G2, and G3 are shown in Figure 5A–C, respectively. In general, there were more UR hub genes than DR hub genes in all three groups. However, in G1, which is the colorectal adenoma stage, we see that 35% of hub genes reported were DR (Figure 5A). In carcinoma stages of G2 and G3, we see that almost 99% of hub genes were UR.

**Figure 6:**
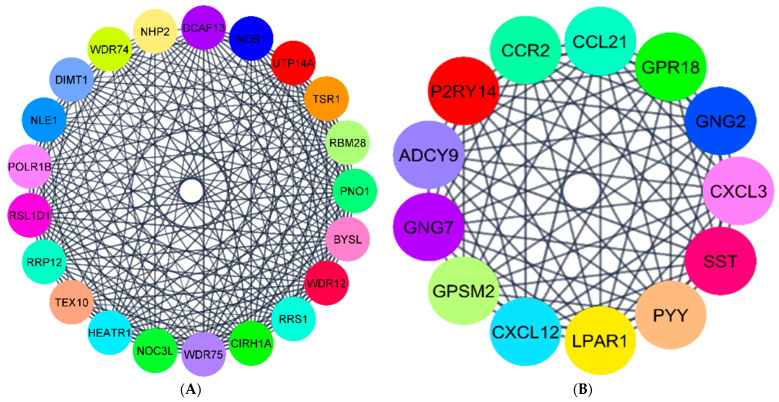
(**A**,**B**) The most significant modules of DEGs in G1. Cluster1 and Cluster2 were obtained with the MCODE plugin in Cytoscape.

Next, we used the MCODE algorithm to build the modular networks and compare the results with the hub genes obtained from the network analyzer method. The top two clusters created with MCODE from the DEGs networks were selected based on their interaction scores. Cluster1 in G1 had 21 nodes and 195 edges (Figure 6A), whereas, cluster2 in G1 had 13 nodes with 77 edges (Figure 6B). These two clusters had all the hub genes determined earlier by the network analyzer method (Figure 5A).

**Figure 7:**
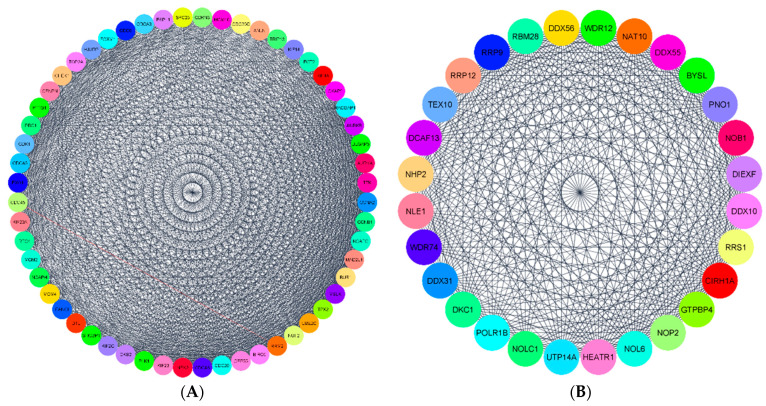
(**A**,**B**) The most significant modules of DEGs in G2. Cluster1 and Cluster2 were obtained with the MCODE plugin in Cytoscape.

The MCODE algorithm generated several clusters in G2, and the top two clusters are shown here. Cluster1 (Figure 7A) in G2 had about 51 nodes and 1078 edges. Cluster2 (Figure 7B) had 23 nodes and 251 edges. These two clusters had all the hub genes determined using the network analyzer algorithm in Figure 5B.

**Figure 8:**
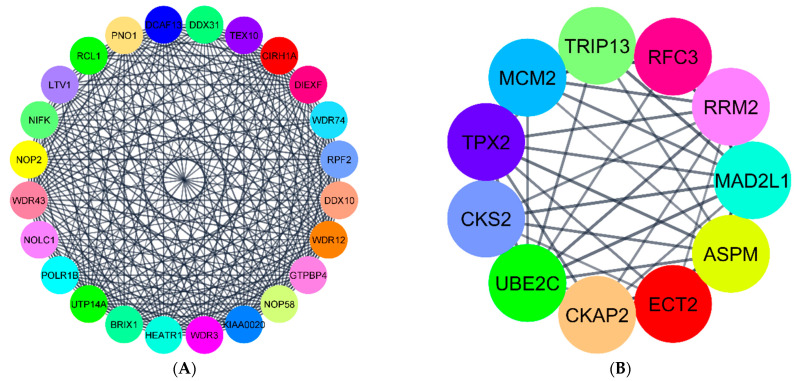
(**A**,**B**) The most significant modules of DEGs in G3. Cluster1 and Cluster2 were obtained with the MCODE plugin in Cytoscape.

For G3 genes, there were a total of 280 nodes and 545 edges in the network. The top two clusters created with MCODE from the PPI network were significant. Cluster1 (Figure 8A) had about 24 nodes and 252 edges. Cluster2 (Figure 8B) had 11 nodes with 43 edges. These clusters had all the hub genes identified by the network analyzer approach reported in Figure 5C.

#### 3.5.2. Functional Enrichment of Hub Genes in KEGG Pathways

The top 40 hub genes in each group are reported in Table S1. The KEGG pathways in which the top 40 hub genes were enriched are shown in Table 4. The ribosome biogenesis in eukaryotes, chemokine signaling pathway, pathways in cancer, and P13K-Akt signaling pathways were the top pathways for the hub genes involved in group G1 (Table 4). CXCL12, CXCL3, and CCL21 hub genes were part of the chemokine signaling pathway.

In G2, the cell cycle, oocyte meiosis, and p53 signaling pathways were highly enriched. Additionally, among the 40 hub genes, 14 were involved in the cell cycle pathway alone in G2. Some of the key hub genes involved in the cell division pathway were CDK1, CDC6, CDC20, CDC45, CHEK1, BUB1, and MAD2L1. The hub genes involved in the p53 signaling pathway were CCNB1, RRM2, CHEK1, and CDK1. Some of the hub genes involved in these two pathways were also involved in oxidative stress and apoptosis. The CDC25C, RRM2, and CDK4 genes were part of the oxidative stress pathway. The hub genes CHEK1, CDK1, CCNB1, and CDC20, were also enriched in the apoptosis pathway.

Finally, ribosome biogenesis in eukaryotes, with a count of nine genes, was the most significantly pathway in G3 (Table 4). Additionally, the hub genes RRM2, POLR1B, POLR1C, and POLR1D were enriched in pyrimidine and purine metabolism pathways in G3. The RRM2 hub gene is also known to be linked to oxidative stress.

We also analyzed the enrichment of 40 hub genes in the GO biological processes for each group, shown in Table S2. We found several key biological processes such as rRNA processing, cell proliferation, and negative regulation of the apoptotic process for G1. For G2, we found that the hub genes were enriched mainly in cell division, mitotic nuclear division, the cell cycle, and the apoptotic process. Finally, for G3, cell division, rRNA processing, positive regulation of gene expression, and cell division were the key biological processes where the hub genes were enriched.

### 3.6. Analyzing DEGs in Oxidative Stress and Apoptosis

As mentioned in the previous section, we found multiple genes in all three groups related to oxidative stress and apoptosis. As a result, we wanted to look into the genes from the different CRC stages that are related to these pathways, in more detail. The dataset description for all the groups and the total count of DEGs found using the Limma procedure for all the datasets from Oncomine are described in Table 2. There are three Oncomine datasets in group-1, seven in group-2, and three in group-3. The total numbers of oxidative stress-related DEGs in Sabates, Skrzypczak, and Skrzypczak2 from group-1 were 29, 24, and 22, respectively. The apoptosis-related DEGs numbered around 200 in these datasets. In group-2, the oxidative stress DEGs ranged from 19 to 39, and the apoptosis DEGs totaled around 200 in all seven Oncomine datasets (Table 2). The number of oxidative stress DEGs in group-3 totaled 16, 28, and 30, respectively, for the datasets Gradudens, Hong, and Skrzypczak2. There were 64, 180, and 188 apoptosis-related DEGs in these datasets. Overall, more DEGs were found in the apoptosis pathway compared to the oxidative stress pathway in all groups. Among the DEGs in both of these pathways, there were more UR than DR genes in all three groups (Table 2).

The RRA algorithm was applied to integrate the list of DEGs from both oxidative stress and apoptosis separately in G1, G2, and G3. The robust integrated list of DEGs established for each group (G1, G2, and G3) related to oxidative stress and apoptosis is noted in Table 5. A *p*-value cut-off of 0.05 was assigned to filter the statistically significant robust DEGs. Relatively fewer robust oxidative stress DEGs were found compared to the number of apoptosis DEGs. More oxidative stress and apoptosis DEGs were found in colorectal adenocarcinoma (G2) than in the other two groups.

The details of robust DEGs involved in the oxidative stress pathway are shown in Table 6. Some of the oxidative stress genes were common among these groups. PRDX6 was present in both G1 and G2. SEPP1 was common between G1 and G3. G2 had the highest number of oxidative stress-related genes compared to the other two groups. The FOXM1 found in G2 (Table 6) was also part of the top 40 hub genes reported in Table S1.

There were more robust DEGs in the apoptosis pathway compared to those in the oxidative stress pathway. Details on gene names and fold change values in each group are shown in Supplementary Table S3. The WDR74 gene is an apoptosis-related gene (Table S3) that was also identified among the top 40 hub genes in G1 (Table S1). CHEK1, CDK1, CCNB1, MCM2, and CDC20 were the apoptosis-related genes in G2 (Table S3). These genes were also found within the top 40 hub genes of G2 (Table S1).

## 4. Discussion

Although the death rate of individuals from CRC has dropped over the past decade, CRC is still the third leading cause of cancer mortalities [1]. CRC is the most common type of malignant tumor among the gastrointestinal tumors, with more than 1.5 million people with a history of CRC in the US [1,30]. New cases are expected to increase to more than 2.2 million worldwide by 2030 [31]. The progression of CRC is a dynamic process, with the expression levels of some molecules changing at different stages [32]. Because of its heterogeneity and complexity, early detection and diagnosis have become increasingly challenging. Effective biomarkers and useful diagnostic approaches for early detection of CRC can improve CRC patient survival rate [33,34]. Therefore, it is necessary to identify meaningful CRC biomarkers and to better understand the molecular mechanisms of CRC progression in different stages [34].

High-throughput technologies, including microarray gene expression and next-generation sequencing, are widely used in cancer research [35]. Microarray data can be used to identify hub genes and pathways related to CRC development [6,11,12,13].

In this study, we focused on expression profiles of normal vs. CRC cases collected from several microarray studies. We included adenoma datasets in addition to carcinomas to examine the differential expression of genes in adenoma when compared to the carcinoma datasets. We divided these datasets into three groups: namely, G1 that included colorectal adenoma vs. normal samples, G2 containing colorectal adenocarcinoma vs. normal samples, and G3 that included colorectal carcinoma vs. normal samples. An RRA approach was applied to integrate the DEGs from multiple datasets into a single list for each group. To better understand the functional roles, we conducted GO enrichment analysis and KEGG pathway analysis for the RRA DEGs. We identified hub genes associated with CRC through computational techniques.

Studies have shown that dysregulation of the cell cycle and mitotic nuclear cell division plays an important role in the occurrence and progression of CRC [8,36,37,38]. We observed that UR DEGs from G1 and G2 were enriched within the cell proliferation and mitotic nuclear division subcategories of the GO biological processes. It was also observed that the rRNA processing and cell division genes were highly enriched in G3 and G2, respectively. The UR DEGs in KEGG pathways showed that the cell cycle, p53 signaling pathway, and microRNAs in cancer pathways were highly enriched in G2 compared to the other groups. The enrichment of cellular response to zinc ion and bicarbonate transport pathways was found in all groups. The bicarbonate transport pathway plays a vital role in diagnosing and treating many cancers, including CRC [6,39]. The KEGG analysis for DR DEGs showed that gene enrichment for the mineral absorption pathway decreased from G1 to G3. The P13K-Akt signaling pathway, bile secretion, and gastric acid secretion were enriched only in G1. Chen et al. (2019) have shown that cancer cell migration and invasion could be suppressed by epithelial-mesenchymal transition (EMT) via P13K/Akt signaling [40].

A PPI network provides a visual framework for better insight into the functional organization of proteins [41]. We used a two-step procedure for generating the PPI networks. The RRA DEGs formed dense networks in each group. The parts of the network that had the highest node interactions were considered and determined to be hub genes using the network analyzer algorithm. The MCODE algorithm was used to obtain clusters that contained significant key genes. The results from the network analyzer, when compared to the results from MCODE for each group, were found to be highly consistent, thereby increasing the reliability. Overall, the top 40 genes with the highest degree of interaction in each group were considered to be hub genes. These hub genes in each group were enriched in several critical pathways related to cancer, as reported in Table 6.

The top KEGG pathways related to hub genes from the adenoma (G1) sets were ribosome biogenesis in eukaryotes, chemokine signaling receptor, pathways in cancer, P13K-Akt signaling pathway, and cytokine-cytokine receptor interaction. The genes involved in the chemokine signaling pathway are CXCL12, CXCL3, and CCL21. CXCL3 has been found to be highly expressed in colorectal adenomas [42,43]. The FOXO1 gene has been implicated to be involved in cancer pathways. Agostini et al. (2008) compared the expression levels of FOXO1 in adenoma and carcinoma tissue and found that the expression levels were significantly higher in adenoma [44]. This result was consistent with our study, where FOXO1 was significantly expressed and was one of the top 40 hub genes in G1. However, it was not significantly expressed in G2 and G3, which are the cancerous stages.

In the adenocarcinoma (G2) datasets, the 40 hub genes were mainly enriched in the cell cycle and p53 signaling pathway. Previous studies have shown that a few of the genes in cell cycle-related pathways promote the proliferation of endothelial cells, contributing to tumor progression and metastasis in colorectal adenocarcinoma [45]. The genes involved in the cell cycle are CDK1, PLK1, TTK, CDC6, CCNA2, CDC20, CCNB1, CDC45, PTTG1, CHEK1, MCM4, BUB1, MCM2, and MAD2L1. The genes involved in the p53 pathway are CDK1, CCNB1, RRM2, and CHEK1. There is support from previously published literature regarding the association of these hub genes in colorectal adenocarcinoma. The CDK1 gene promotes cell proliferation by inhibition of the FOXO1 transcription factor [46]. The effects of alteration of the CDK1 gene are seen in many cancer types, including esophageal adenocarcinoma, breast cancer, oral squamous cell carcinoma, and hepatocellular and pancreatic adenocarcinoma [47,48,49,50,51]. Lu et al. (2012) have demonstrated that the RRM2 gene is overexpressed in colorectal adenocarcinoma, and the higher expression of RRM2 was correlated with the tumor node metastasis stage [52]. Gan et al. (2018) revealed that the expression of the CCNA2 gene is higher in colorectal adenocarcinoma tissues than in normal samples and showed that knockdown of CCNA2 could suppress cell growth by disrupting the cell cycle and inducing cell apoptosis [53]. Takahashi et al. (2003) showed that PLK1 is overexpressed in CRC [54]. PLK1 is also involved in the proliferation, migration, and invasion of CRC cells [55]. Gali-Muhtasib et al. (2008) proved that overexpression of CHEK1 was correlated with advanced tumor stages, with worse prognosis in CRC [56]. There are a few other hub genes in G2 that are not a part of the cell cycle and p53 pathways. MAD2L1 and BUB1 are critical components of the spindle assembly [57] and are known to be important drivers of carcinogenesis in colorectal adenocarcinoma [58,59]. Ding et al. (2020) demonstrated that cell growth suppression by knockdown of MAD2L1 impaired cell cycle progression and induced cell apoptosis [3]. The AURKA and TPX2 genes are also related to carcinogenesis in CRC by promoting the progression of colorectal adenoma to colorectal adenocarcinoma [60]. Ren et al. (2017) revealed that the downregulation of PTTG1 suppressed cell proliferation and invasion in CRC [61]. The DTL gene is known for its role in cell proliferation and cell cycle arrest in many cancers such as breast [62], hepatocellular carcinoma [63], and gastric cancer [64]. Whereas NUSAP1 plays an essential role in mitotic spindle assembly [65],. it has been found to induce apoptosis and inhibit proliferation, migration, and invasion in CRC [66]. KIF11 is another protein required for spindle formation [67]. The knockdown of KIF11 prevents sphere formation, indicating its importance in CRC [68]. The TOP2A gene causes chromosomal instability in many different cancers [69,70,71], and its protein expression level is linked to advanced tumor stages and chemotherapeutic resistance via inhibition of apoptosis [72]. CEP55 enhances cell growth, and its knockdown inhibits cell growth in breast and gastric cancers [73,74].

The top 40 hub genes detected from colorectal carcinoma (G3) datasets were found to be related to ribosome biogenesis in eukaryotes, rRNA processing, and pyrimidine and purine metabolism pathways. Previous studies have shown that the ribosome biogenesis pathway is altered in CRC, and the alterations lead to increased production of ribosomes linked to the initiation and progression of colorectal carcinogenesis [75]. Stedman et al. (2015) showed that the dysfunction of ribosome biogenesis might also lead to p53-mediated apoptosis in some cancers [76].

We further examined the presence of the oxidative stress and apoptosis pathway genes related to CRC in the Oncomine database. FOXM1, CDC25C, RRM2, CDK4, PRDX1, PRDX2, GPX2, GPX4, and FHL2 genes were the core genes found to be related to the oxidative stress pathway. The FOXM1 and RRM2 genes were also found in the cell cycle and p53 signaling pathway. Slattery et al. (2019) have suggested that the activation of p53 signaling may be related to cellular stress, which could influence apoptosis, cell cycle arrest and angiogenesis through the mRNA–miRNA interactions [77]. The PRDX1 and PRDX2 genes regulate cellular signaling and differentiation. These genes were found to be upregulated in our analysis and may serve as potential therapeutic targets. Previous studies have also shown that these genes are upregulated and promote metastasis and angiogenesis in CRC [78,79]. Verset et al. (2013) have demonstrated that higher expression of FHL2 is involved in the progression of CRC [80].

The genes linked to the apoptosis pathway were CHEK1, CDK1, CCNB1, GTSE1, MCM2, MCM10, CDC20, WDR74, and PMAIP1. As described in one of the previous sections, CHEK1, CDK1, and CCNB1 were involved in p53 signaling. Our analysis demonstrates that these are some of the major genes related to apoptosis pathways. The tumors lacking p53 have shown higher levels of expression of CHEK1, which was accompanied by inability to induce apoptosis [56]. The higher expression of CDK1 and CDC20 in CRC patients is associated with a poorer prognosis [81]. The GTSE1 gene promotes cell growth in breast cancer by activating the P13-Akt pathway and enhances metastasis. It can also regulate the p53 to alter the cell cycle [82]. The expression of PMAIP1, also known as the NOXA gene, is regulated by p53 signaling and has been found to be involved in p53-mediated apoptosis [83].

As future work, we plan to validate the differential expression of the target hub genes in commercially available cell lines by qRT-PCR. We also plan to examine the expression of these target transcripts during the different stages of CRC. The study of the genes in the key pathways related to tumorigenesis could lead to targets for novel therapeutic interventions.

## 5. Conclusions

In this study, we used multiple GEO datasets for different CRC types and found 40 hub genes from each group through various bioinformatics approaches. The hub genes were found to be enriched in the cell cycle, p53 signaling pathway, mineral absorption, and many other key pathways related to CRC progression. We also analyzed the genes involved in stress-survival and apoptosis-related pathways using the Oncomine database. Our findings suggest that the hub genes revealed in our study may be considered as biomarkers for the diagnosis of CRC. Additionally, the genes expressed in stress-survival pathways could be tested as potential therapeutic targets. However, this study, as a primarily in-silico analysis, had its limitations; in vivo and in vitro experiments would be needed to validate the biological functions of these genes in CRC.

## Figures and Tables

**Table 1:** Description of the datasets used in the study.

Groups	Dataset	# Samples (Cases/Control)	# Genes	Platform	Source	DEGs (UR/DR)
Group-1 (CA)	GSE8671	64 (32/32)	54675	GPL570	[15]	5498 (1355/4143)
	GSE20916	69 (45/24)	27697	GPL570	[16]	2761 (1290/1471)
	GSE89076	80 (41/39)	58717	GPL16699	[17]	7538 (2844/4694)
Group-2 (CAC)	GSE20842	130 (65/65)	40645	GPL4133	[18]	3112 (1432/1680)
	GSE20916	60 (36/24)	27697	GPL570	[16]	3556 (1820/1736)
	GSE39582	585 (566/19)	54675	GPL570	[19]	3389 (1582/1807)
	GSE110225	34 (17/17)	54675	GPL570	[20]	1109 (478/631)
	TCGA-COAD	519 (478/41)	56499	Illumina	[21]	6703 (3180/3523)
	TCGA-READ	176 (166/10)	56493	Illumina	[22]	6813 (3291/3522)
Group-3 (CC)	GSE3964	30 (18/12)	23232	GPL3282	[23]	483 (158/325)
	GSE113513	28 (14/14)	49395	GPL15207	Unpublished	2864 (1151/1713)
	GSE32323	44 (17/27)	54675	GPL570	[24]	4671 (4530/141)
	GSE21510	148 (123/25)	54675	GPL570	[25]	7720 (4383/3137)

Note: CA—Colorectal adenoma; CAC—Colorectal adenocarcinoma; CC—Colorectal carcinoma; DEGs—Differentially expressed genes; UR—Upregulated; DR—Downregulated; #—Total count.

**Table 2:** Description of the Oncomine datasets used in this study.

Oncomine Groups	Dataset	# Samples (Cases/Control)	Oxidative Stress DEGs (UR/DR)	Apoptosis DEGs (UR/DR)
Oncomine Group-1 (CA)	Sabates (GSE8671)	64 (32/32)	29 (21/08)	208 (142/66)
	Skrzypczak (GSE20916)	69 (45/24)	24 (15/09)	189 (105/84)
	Skrzypczak2 (GSE20916)	15 (5/10)	22 (12/10)	176 (90/86)
Oncomine Group-2 (CAC)	Dulak (GSE36458)	122 (62/60)	19 (10/09)	175 (93/82)
	Gaedcke (GSE20842)	130 (65/65)	39 (23/16)	199 (107/92)
	Kaiser (GSE5206)	54 (49/5)	37 (20/17)	225 (117/108)
	Kurashina (GSE11417)	184 (94/90)	29 (18/11)	173 (111/62)
	Skrzypczak (GSE20916)	60 (36/24)	27 (15/12)	187 (101/86)
	TCGA CRC	184 (162/22)	38 (21/17)	257 (120/137)
	TCGA CRC2	970 (389/581)	33 (17/16)	209 (108/101)
Oncomine Group-3 (CC)	Graudens (GSE3964)	30 (18/12)	16 (09/07)	64 (20/44)
	Hong (GSE9348)	82 (70/12)	28 (16/12)	180 (110/70)
	Skrzypczak2 (GSE20916)	15 (5/10)	30 (15/15)	188 (97/91)

Note: CA—Colorectal adenoma; CAC—Colorectal adenocarcinoma; CC—Colorectal carcinoma; DEGs—Differentially expressed.genes; UR—Upregulated; DR—Downregulated; #—Total count.

**Table 3:** Robust DEGs (*p*-value < 0.05) in each group from Table 1.

Groups	Robust UR	Robust DR	Total Robust DEGs
G1 (CA)	186	449	635
G2 (CAC)	499	494	993
G3 (CC)	206	79	285

**Table 4:** KEGG Pathways related to hub genes.

Group	Term	KEGG Pathway	Count	*p*-Value	Genes
G1	hsa03008	Ribosome biogenesis in eukaryotes	7	2.92 × 10^−7^	RBM28, NOB1, HEATR1, NHP2, WDR75, UTP14A, RAN
	hsa04062	Chemokine signaling pathway	7	2.48 × 10^−5^	CXCL12, ADCY9, GNG2, CCL21, GNG7, CXCL3, CCR2
	hsa05200	Pathways in cancer	8	0.000208	CXCL12, ADCY9, GNG2, MYC, GNG7, LPAR1, IGF1, FOXO1
	hsa04151	PI3K-Akt signaling pathway	5	0.025838	GNG2, MYC, GNG7, LPAR1, IGF1
	hsa04727	GABAergic synapse	3	0.032272	ADCY9, GNG2, GNG7
	hsa05032	Morphine addiction	3	0.036574	ADCY9, GNG2, GNG7
	hsa04713	Circadian entrainment	3	0.03956	ADCY9, GNG2, GNG7
	hsa04723	Retrograde endocannabinoid signaling	3	0.044207	ADCY9, GNG2, GNG7
	hsa04060	Cytokine-cytokine receptor interaction	4	0.045786	CXCL12, CCL21, CXCL3, CCR2
G2	hsa04110	Cell cycle	14	2.75 × 10^−19^	PLK1, TTK, CDC6, CCNA2, CDC20, CCNB1, CDC45, PTTG1, CHEK1, CDK1, MCM4, BUB1, MCM2, MAD2L1
	hsa04114	Oocyte meiosis	8	1.01 × 10^−8^	CDC20, CCNB1, PTTG1, PLK1, CDK1, BUB1, MAD2L1, AURKA
	hsa04914	Progesterone-mediated oocyte maturation	6	2.91 × 10^−6^	CCNA2, CCNB1, PLK1, CDK1, BUB1, MAD2L1
	hsa04115	p53 signaling pathway	4	0.000765	CCNB1, RRM2, CHEK1, CDK1
	hsa05203	Viral carcinogenesis	4	0.01777	CCNA2, CDC20, CHEK1, CDK1
	hsa05166	HTLV-I infection	4	0.031122	CDC20, PTTG1, CHEK1, MAD2L1
G3	hsa03008	Ribosome biogenesis in eukaryotes	9	1.05 × 10^−11^	RCL1, NOP58, WDR3, HEATR1, RPP40, UTP14A, GTPBP4, WDR43, RAN
	hsa00240	Pyrimidine metabolism	4	0.001799	RRM2, POLR1B, POLR1C, POLR1D
	hsa03020	RNA polymerase	3	0.00273	POLR1B, POLR1C, POLR1D
	hsa00230	Purine metabolism	4	0.008596	RRM2, POLR1B, POLR1C, POLR1D

**Table 5:** Robust DEGs related to oxidative stress and apoptosis genes in all groups from Oncomine.

Groups	Robust Oxidative stress DEGs (UR/DR)	Robust Apoptosis DEGs (UR/DR)
G1	04 (02/02)	28 (19/09)
G2	09 (04/05)	47 (23/24)
G3	02 (00/02)	18 (09/09)

**Table 6:** Robust oxidative stress-related DEGs from Oncomine.

Groups	Robust Oxidative-Stress DEGs	*p*-Value	Fold Change
G1	GPX2	4.95 × 10^−6^	2.282463
	KIF9	3.95 × 10^−6^	2.274907
	PRDX6	1.23 × 10^−7^	−2.7178
	SEPP1	0.000103	−4.44091
G2	FOXM1	0.009917	1.598071
	GSS	0.00024	1.346084
	NUDT1	0.000948	1.576259
	PRDX2	0.000454	1.372055
	ANGPTL7	0.005649	−4.24904
	MSRA	7.11 × 10^−5^	−1.59741
	PDLIM1	0.002234	−1.33504
	PRDX6	0.00074	−2.2563
	SCARA3	0.000324	−1.59527
G3	CCL5	2.39 × 10^−5^	−1.84543
	SEPP1	8.97 × 10^−7^	−4.10094

## Data Availability

All the GEO datasets reported in this study were obtained from the https://www.ncbi.nlm.nih.gov/geo/, accessed on 24 August 2020. database. These datasets can be retrieved with the GEO accession numbers. The TCGA datasets were obtained from the https://portal.gdc.cancer.gov/, accessed on 24 August 2020 portal. The Oncomine datasets were obtained from https://www.oncomine.org/, accessed on 7 May 2020 database.

## Supplementary Materials

The following are available online at https://www.mdpi.com/article/10.3390/ijerph18115564/s1, Figure S1: Heatmap showing the expression levels of robust DEGs across all groups, Figure S2: PPI network for G1, Figure S3: PPI network for G2, Figure S4: PPI network for G3, Table S1: Top forty hub genes in each group, Table S2: GO enrichment analysis for top forty hub genes, and Table S3: Apoptosis related genes.
