# Supplementary figures and images for: Identification of Hub Genes in Different Stages of Colorectal Cancer through an Integrated Bioinformatics Approach
Source: Int J Environ Res Public Health. 2021 May 23;18(11):5564. doi: 10.3390/ijerph18115564 (PMC8197092; doi:10.3390/ijerph18115564)

Figure S1: Heatmap showing the expression levels of robust DEGs across all groups

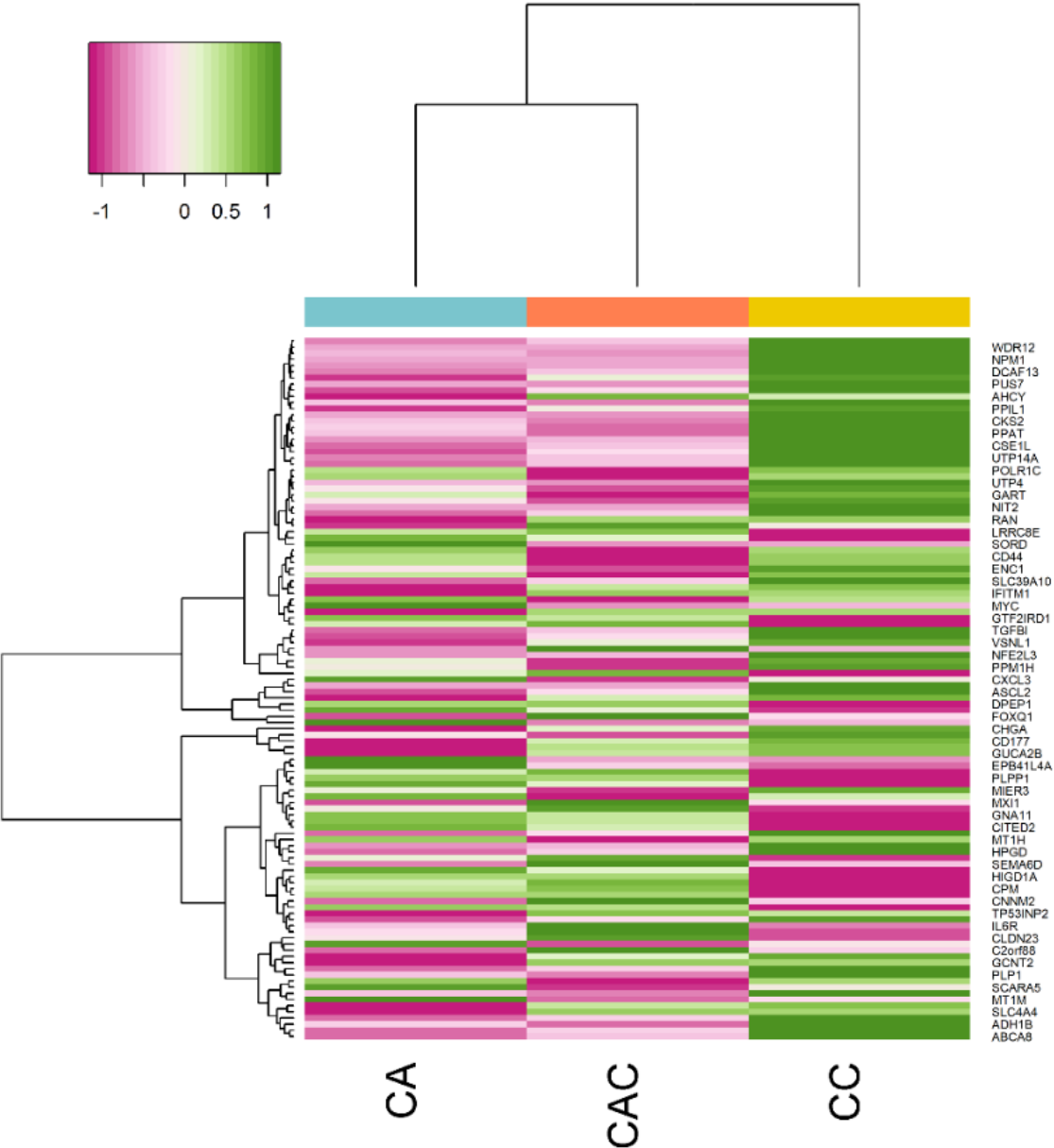

Supplement: Supplementary file 1 [file ijerph-18-05564-s001.zip › Figure S1.pdf]

Figure S2: PPI network of DEGs from G1 constructed in Cytoscape with String-db.

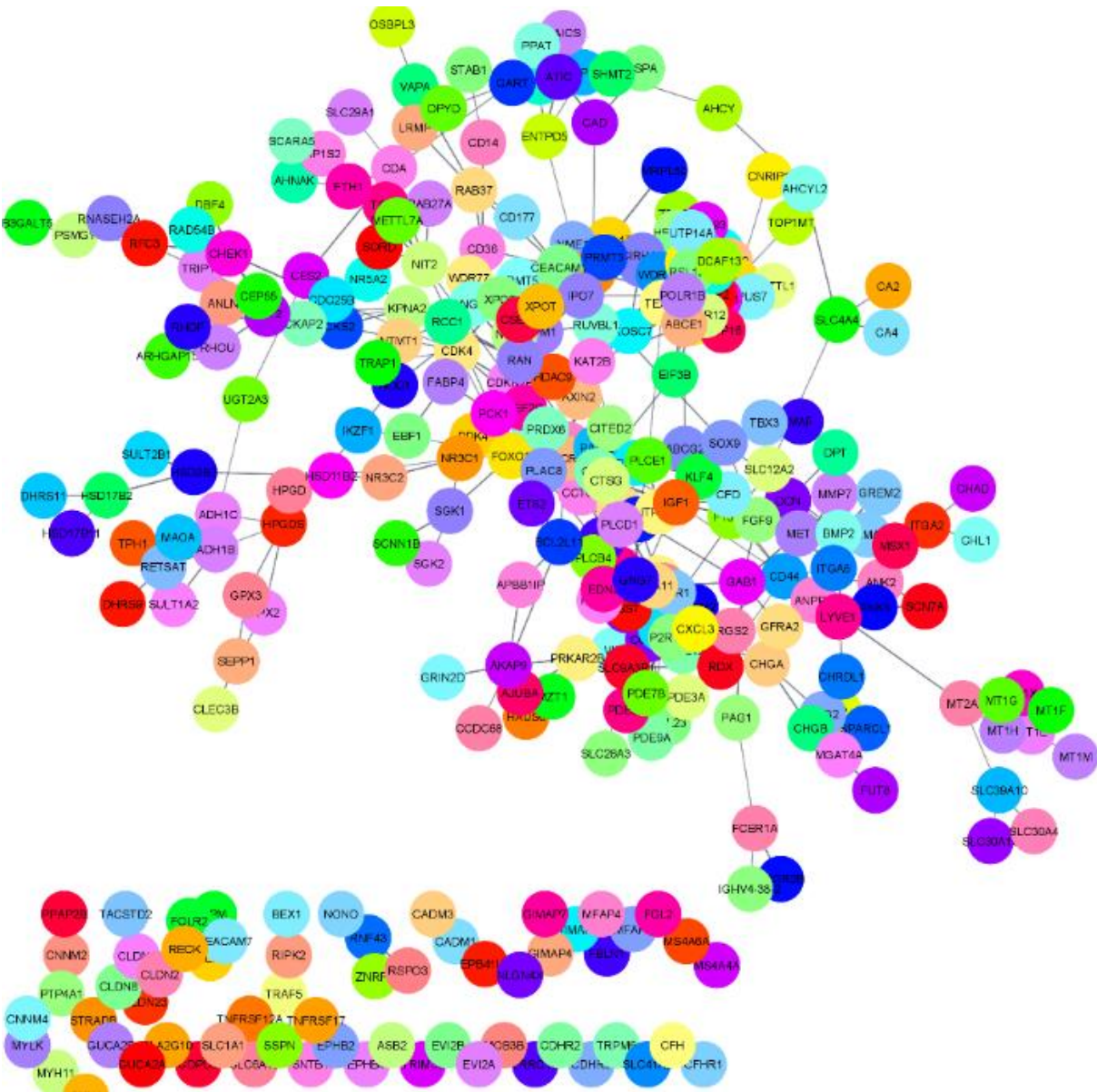

Supplement: Supplementary file 1 [file ijerph-18-05564-s001.zip › Figure S2.pdf]

Figure S3: PPI network of DEGs from G2 constructed in Cytoscape with String-db.

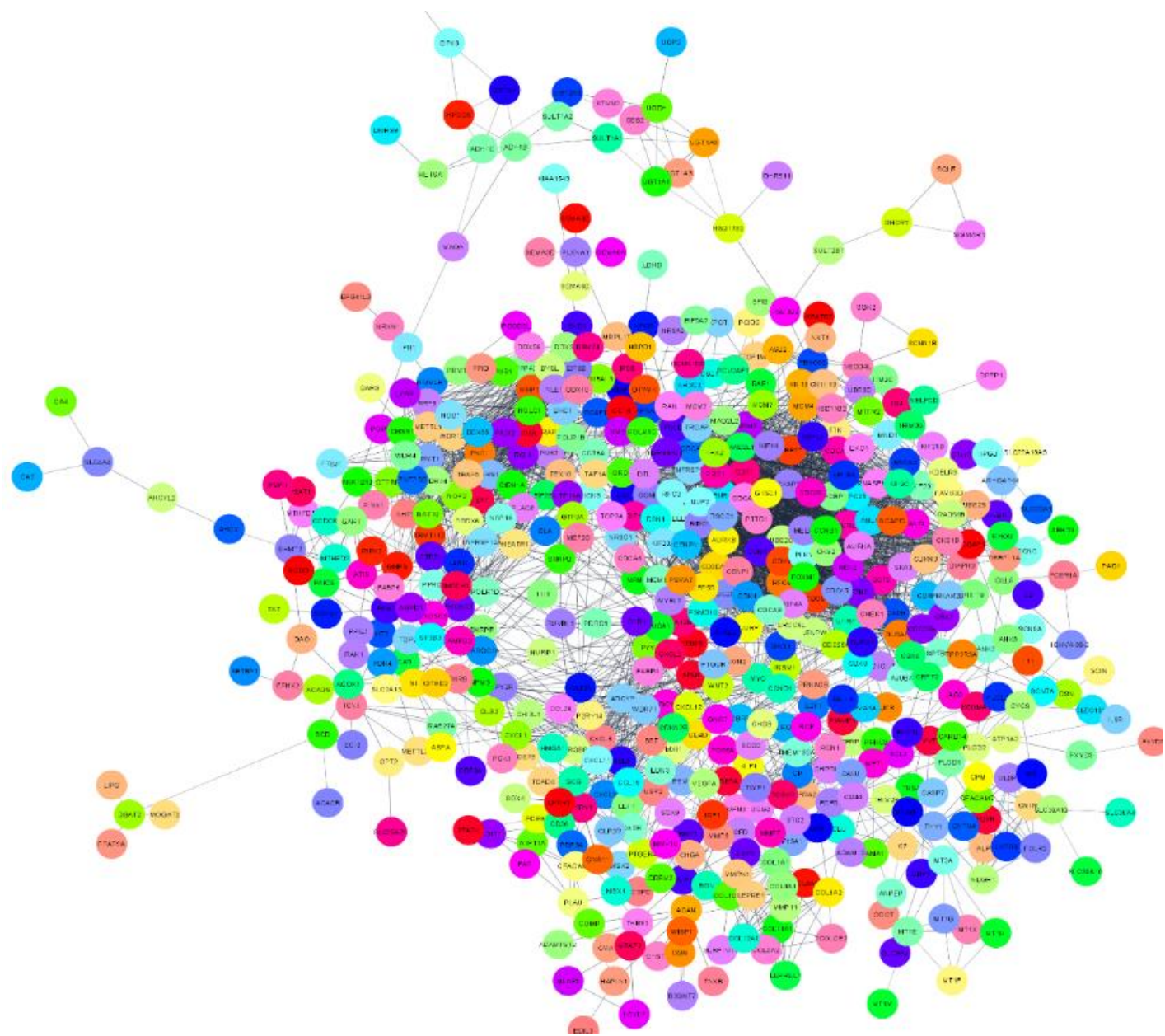

Supplement: Supplementary file 1 [file ijerph-18-05564-s001.zip › Figure S3.pdf]

Figure S4: PPI network of DEGs from G3 constructed in Cytoscape with String-db.

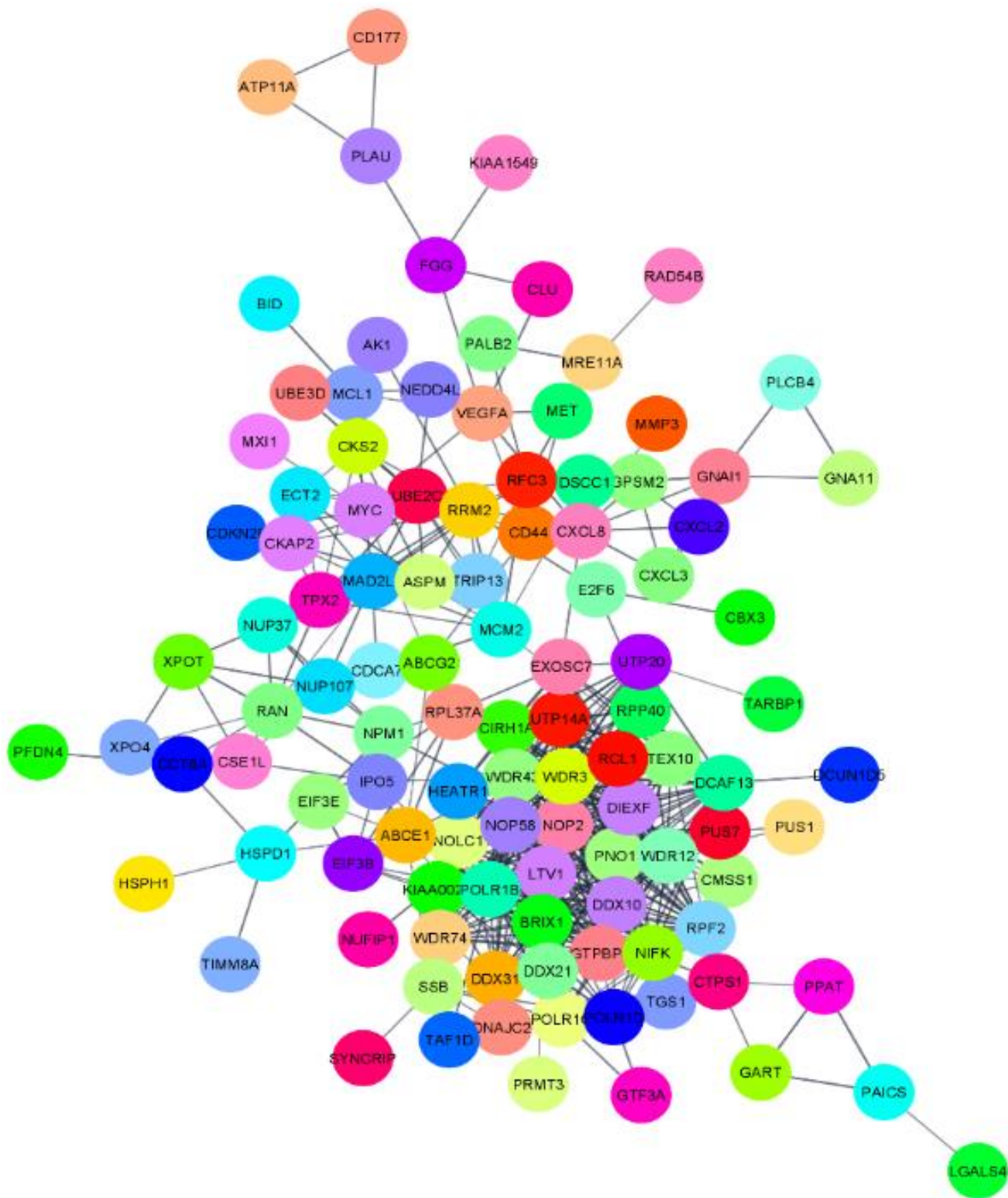

Supplement: Supplementary file 1 [file ijerph-18-05564-s001.zip › Figure S4.pdf]
